# Supplementary material for: The energetics of rapid cellular mechanotransduction
Source: Proc Natl Acad Sci U S A. 2023 Feb 16;120(8):e2215747120. doi: 10.1073/pnas.2215747120 (PMC9974467; doi:10.1073/pnas.2215747120)
Supplement: Supplementary file 1 — Appendix 01 (PDF) [file pnas.2215747120.sapp.pdf]

PNAS

Supporting Information for

The energetics of rapid cellular mechanotransduction

Michael N. Young[1], Michael J. Sindoni[1], Amanda H. Lewis[1], Stefan Zauscher[2], and Jörg Grandl[1,*]

*Correspondence: Jorg Grandl

Email: grandl@neuro.duke.edu

This PDF file includes:

Figures S1 to S7

Figure S1.

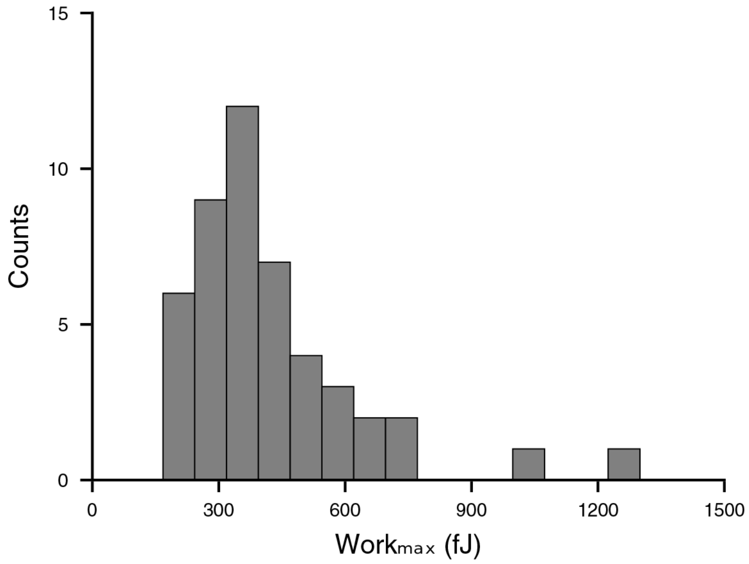

Figure S1. Maximum work applied to Piezo1-expressing HEK293T cells.

Histogram showing the distribution of peak works applied across all Piezo1-expressing cells.

Figure S2.

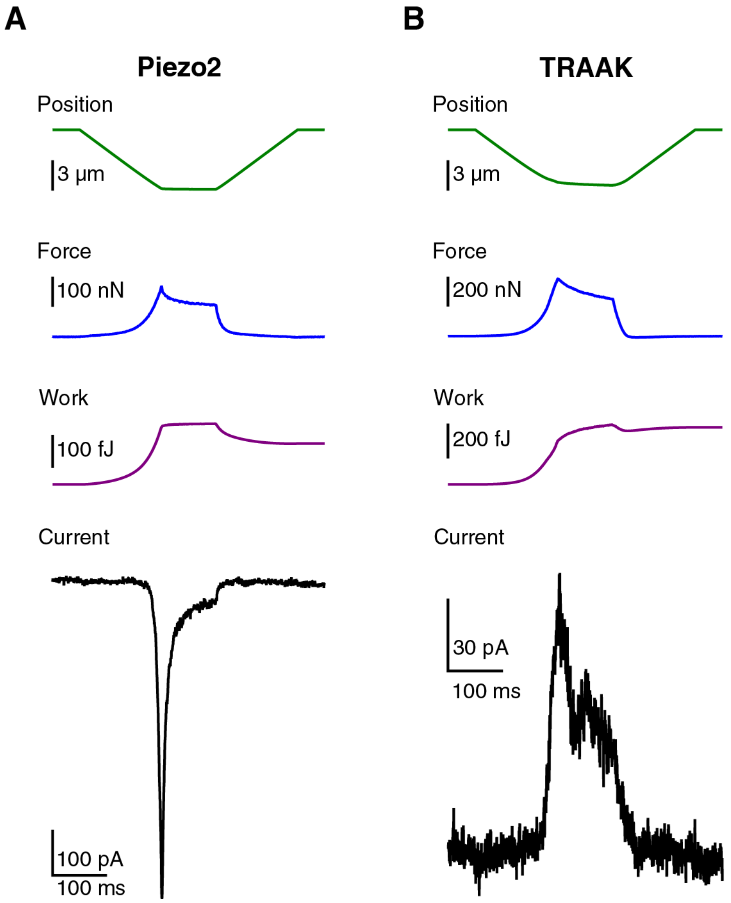

Figure S2. Representative Traces for Piezo2, TRAAK, and TREK1.

(A) Representative trace for Piezo2 at -80 mV. **(B)** Representative trace for TRAAK at 0 mV. **(C)** Representative trace for TREK1 at 0 mV.

Figure S3.

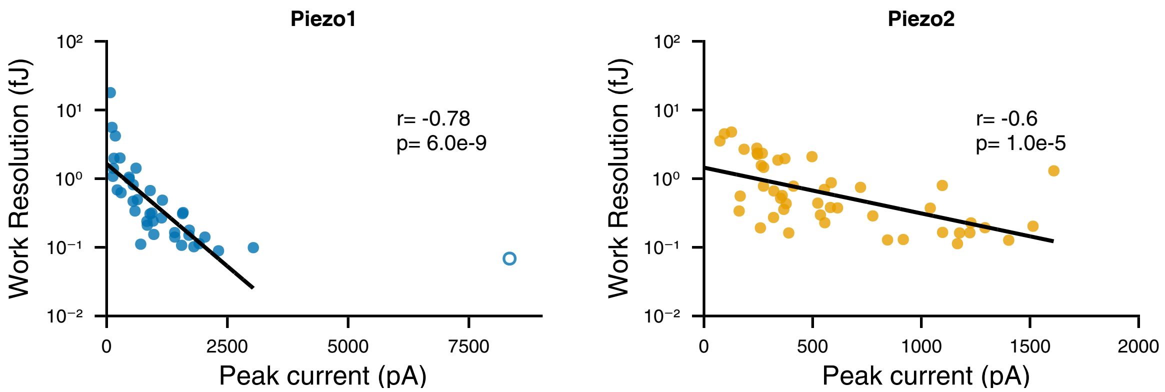

Figure S3. Work resolution correlates with peak current amplitude.

Correlation between values for work resolution and peak current for Piezo1 (blue), Piezo2 (orange). Work resolution is represented on a logarithmic scale. Black lines are linear fits to the data. Correlation coefficients and p-values are indicated on the top left.

Figure S4.

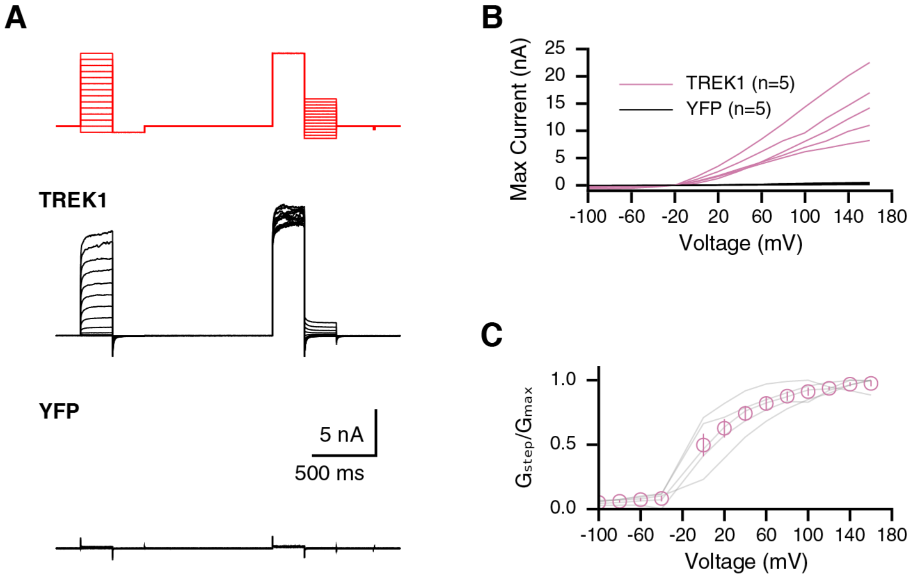

Figure S4. TREK1 voltage-dependence saturates in Rb^+ internal solution.

(A) Voltage-step and tail-current protocol and representative traces of cells overexpressing TREK1 or YFP. **(B)** Individual current-voltage relationships from cells expressing TREK1 (pink) or YFP (black). N indicates the number of replicates (individual cells). **(C)** Normalized average conductance-voltage relationship for cells shown in B.

Figure S5.

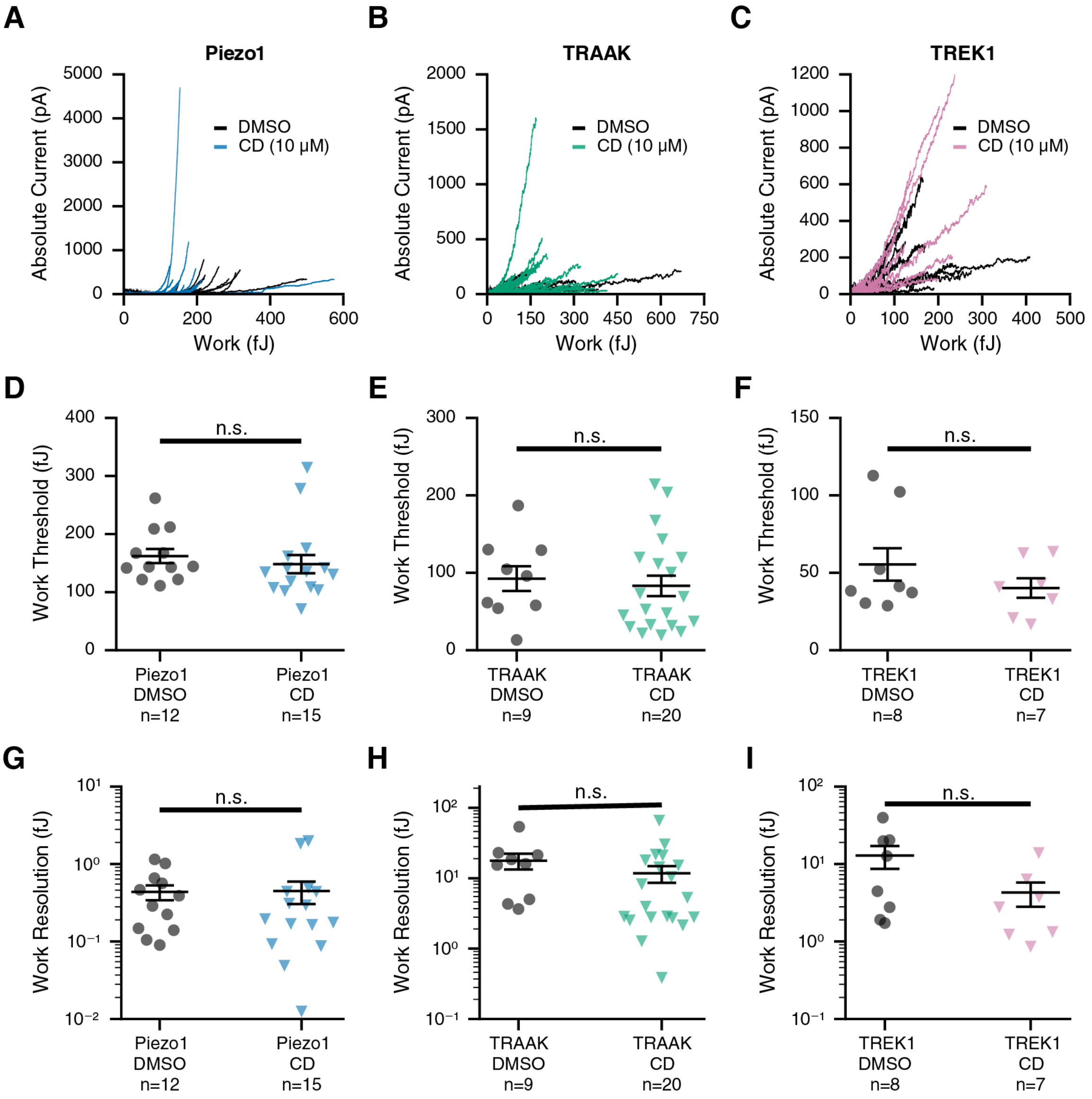

Figure S5. Piezo1, TRAAK, and TREK1 detection threshold and resolution are insensitive to Cytochalasin D treatment.

Representative work-current relationships for Piezo1 **(A),** TRAAK **(B),** and TREK1 **(C)**. DMSO-treated control cells are shown in black, and cells treated with 10 μM Cytochalasin D are shown in blue, green, or pink respectively. Work threshold values for Piezo1 **(D)**, TRAAK **(E)**, and TREK1 **(F)**. Work resolution values for Piezo1 **(G)**, TRAAK **(H)**, and TREK1 **(I)**. Colors for D-I are as in A-C. For D-I, lines indicate the bootstrapped mean and SEM. N indicates the number of replicates (individual cells). Group comparisons in D-I were performed using Welch's T-test.

Figure S6.

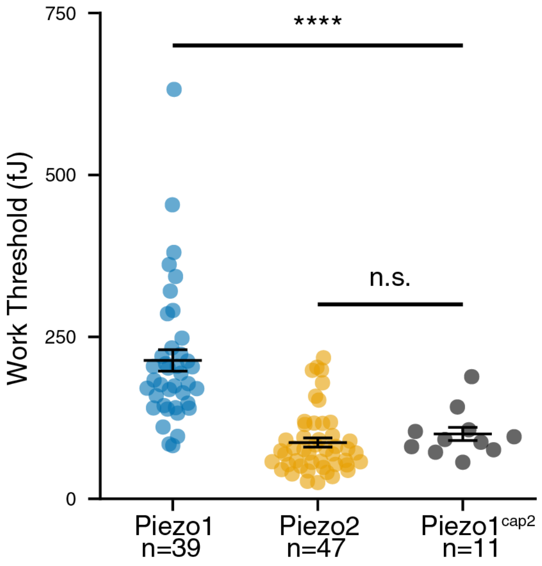

Figure S6. The cap domain confers the work threshold for Piezo ion channels.

Work threshold values for cells overexpressing Piezo1 (blue), Piezo2 (orange), and chimera Piezo1^{cap2} (black). N indicates the number of replicates (individual cells). Group-wise comparisons were performed using Welch's ANOVA followed by a post-hoc Games-Howell test (adjusted p-values for Piezo1-Piezo1^{cap2}:4.06e-6 and Piezo2-Piezo1^{cap2}:0.591).

Figure S7.

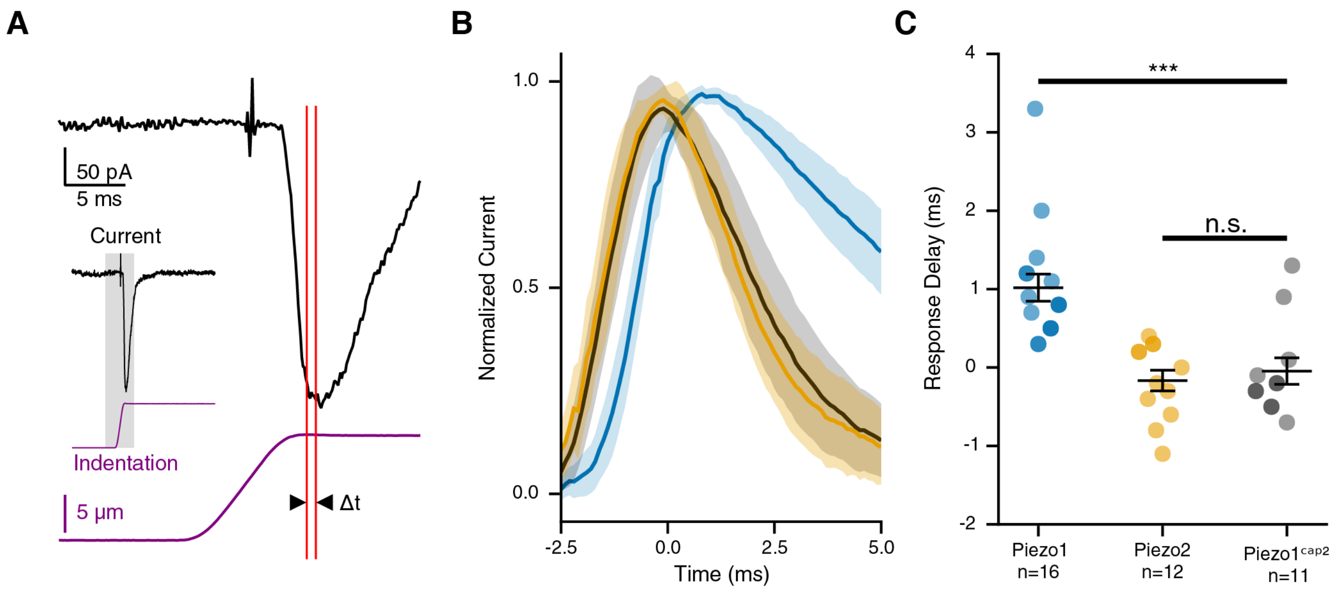

Figure S7. Cells expressing Piezo1 show a response delay in whole-cell poke stimulation. **(A)** Zoomed-in representative poke current trace (black) and stimulus (purple). Response delay (Δt) was determined based on the time difference between the peak of the current response and the end of the actuator displacement both shown in red). A zoomed-out view of the trace is shown in the inset with the region of interest highlighted in gray. **(B)** Average current traces upon whole-cell poke stimulation for cells overexpressing Piezo1 (blue), Piezo2 (orange), and chimera Piezo1^{cap2} (black). Traces were normalized to their peak and aligned to the end of the end of the actuator displacement of the poke stimulation. Bands are 95% confidence intervals. **(C)** Response delay values, reflecting the time difference between the stop of the poke stimulation and the peak of the transduction current, for cells overexpressing Piezo1 (blue), Piezo2 (orange), and chimera Piezo1^{cap2}. N indicates the number of replicates (individual cells). Group-wise comparisons were performed using Welch's ANOVA followed by a post-hoc Games-Howell test (adjusted p-values for Piezo1-Piezo1^{cap2}:0.001 and Piezo2-Piezo1^{cap2}:0.861).
